# Progression of planovalgus deformity in patients with cerebral palsy

**DOI:** 10.1186/s12891-020-3149-0

**Published:** 2020-03-03

**Authors:** Jae Jung Min, Soon-Sun Kwon, Ki Hyuk Sung, Kyoung Min Lee, Chin Youb Chung, Moon Seok Park

**Affiliations:** 1 Department of Orthopedic Surgery, Seoul National University Bundang Hospital, 82 Gumi-ro 173 Beon-gil, Bundang-Gu, Sungnam, Gyeonggi 13620 South Korea; 2 Department of Mathematics, College of Natural Sciences, Ajou University, Gyeonggi, South Korea

**Keywords:** Pes planovalgus, Talo-first metatarsal angle, Talo-second metatarsal angle, Progression, Cerebral palsy

## Abstract

**Background:**

Analyzing radiographic changes of pes planovalgus(PV) deformity of cerebral palsy(CP) patients according to age and influencing factors.

**Methods:**

CP patients with PV deformity younger than 18 years old who had undergone more than a year of follow-up with at least two standing foot radiographs were included. Anteroposterior and lateral talo-first metatarsal(talo-1stMT), talo-second metatarsal(talo-2ndMT), and hallux valgus(HV) angles were measured on the radiographs. The rate of progression was adjusted by multiple factors using the linear mixed model, with the Gross Motor Function Classification System(GMFCS) level as the fixed effect and age and each subject as random effects.

**Results:**

Overall, 194 patients were enrolled in this study, and 1272 standing foot radiographs were evaluated. The AP talo-2^nd^MT angle progressed by 0.59° (*p* < 0.0001) and 0.64° (*p* = 0.0007) in GMFCS level II and III patients, respectively; however, there was no significant change in GMFCS level I patients (*p* = 0.3269). HV was significantly affected by age in all three GMFCS groups; it increased by 0.48° (*p* < 0.0001), 0.66° (p < 0.0001), and 1.19° (p < 0.0001) for levels I, II, and III, respectively. The lateral talo-1stMT angle showed improvements in GMFCS level I and II patients (0.43°, *p* < 0.0001, and 0.61°, p < 0.0001, respectively). In GMFCS level III patients, there was no significant improvement in the lateral talo-1^st^MT angle (*p* = 0.0535).

**Conclusions:**

The GMFCS level was the single most important factor influencing the progression of radiographic indices in PV deformity in CP. The AP talo-1^st^MT and talo-2ndMT angles progressed in patients with GMFCS levels II and III. Physicians should take this result into consideration when planning the timing of the surgery.

**Level of evidence:**

Prognostic Level IV.

## Background

Pes planovalgus(PV) is one of the most common foot deformities in patients with cerebral palsy(CP), especially in patients with diplegia and quadriplegia [1]. PV in CP most likely results from muscle imbalance and spasticity in a skeletally developing foot [2]. Due to prominent talar head, PV frequently leads to pain during weight-bearing. Furthermore, in patients with CP, PV results in lever-arm dysfunction [3]: not only is PV a prototype of a flexible lever arm, but it also causes malrotated lever-arm dysfunction by shortening foot lever due to the externally rotated or abducted forefoot [3–6]. In patients who already have weak muscles, lever-arm dysfunctions further worsen their ambulatory function [3].

Idiopathic PV in children and adolescents improves as patients grow older [7–9], as seen in cross-sectional studies [8, 9], and its spontaneous recovery has also been proven radiographically in a longitudinal study [7]. The anteroposterior(AP) talo-1stMT and lateral talo-1stMT angles improved annually until skeletal maturity [7]. However, PV deformity in CP may not follow this pattern, since underlying condition does not disappear. While spasticity continuously affects the extremities, forefoot dragging due to malrotated lever and Achilles contractures may further interrupt spontaneous improvement observed in idiopathic PV [5]. Also, recurrence of PV after calcaneal lengthening is greater in GMFCS III and IV patients, implying that PV can progress even after the surgical procedure [10]. However, there are few studies that invistigated progression of PV in CP patients.

Therefore, the purpose of this study was to evaluate the trend in radiologic changes of CP patients with PV deformity according to age and identify influencing factors.

## Methods

### Patients and study design

This retrospective study was approved by the institutional review board of tertiary referral center for CP (IRB no. B-1905-541-106). Informed consent was waived due to retrospective nature of the study.

We screened patients seen between May 2003 and May 2019 via clinical data warehouse to identify those with CP who were ≤ 18 years of age, who had been followed up for more than 1 year, with at least two standing AP and lateral foot radiographs.

**Fig. 1:**
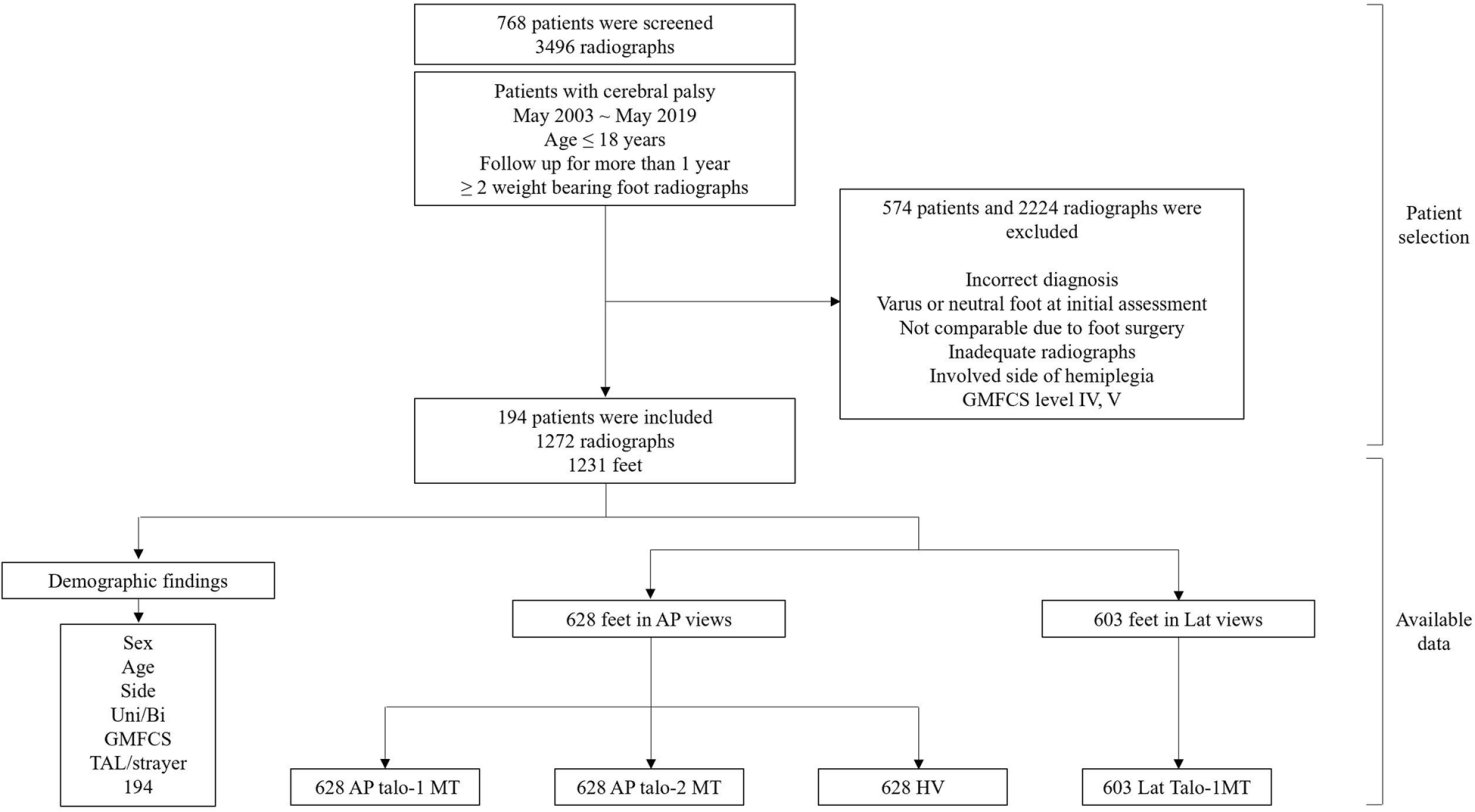
Inclusion and exclusion criteria. GMFCS, Gross Motor Function Classification System; Uni, unilateral; Bi, bilateral; TAL, tendo-Achilles lengthening; AP, anteroposterior; Lat, lateral; AP talo-1MT, AP talo-first metatarsal angle; AP talo-2MT, AP talo-second metatarsal angle; HV, hallux valgus angle; Lat talo-1MT, lateral talo-first metatarsal angle

After screening, two authors(JJM and MSP) reviewed and screened medical records and radiographs. The exclusion criteria were (1)incorrect diagnosis, (2)varus or neutral foot at initial presentation i.e., talo-2^nd^MT angle< 10°, (3)inadequate radiographs, such as inadequate weight-bearing, (4)involved side of hemiplegia, and (5)GMFCS levels IV and V. For those who had undergone intervention for foot deformity, such as calcaneal lengthening or talonavicular fusion, postsurgical radiographs taken on the operated side were excluded (Fig. 1). Standing AP and lateral foot radiographs were taken with a UT 2000 X-ray machine(Philips Research, Eindhoven, The Netherlands) at a source-to-image distance of approximately 100 cm with the patient standing barefoot. The X-ray machine settings ranged from 46 to 50 kVp and 4.5 to 5 mAs, according to each patients’ body size. All radiographic images were digitally acquired with the use of a picture archiving and communication system(PACS) (INFINITT Healthcare, Seoul, Korea), and measurements were subsequently carried out with the use of PACS software.

### Consensus building and measurement

Five authors(JJM, KHS, KML, and MSP, who are orthopedic surgeons with 2, 16, 17, and 19 years of experience, respectively, and S-SK, a statistician) agreed on the indices to be measured in the radiographs. Previous studies were reviewed [7, 11–15], and one of the authors(JJM) pooled seven indices relevant to the PV and hallux valgus(HV) that have been established with convergent and discriminant validities and intra, inter-rater reliabilities [12]: naviculocuboid(NC) overlap, AP talonavicular(TN) coverage angle, AP and lateral talo-1^st^MT angles, AP talo-2^nd^MT angles, HV angle, and intermetatarsal angle. In our study, NC overlap and AP TN coverage angle were excluded due to incomplete ossification of the navicular bones in younger patients, making evaluation of indices that include the navicular bone difficult. We have also noted that not only does PV progress over time, but HV also progressed in CP patients, which may disturb the talo-1^st^MT angle. Therefore, we introduced the talo-2^nd^MT angle as a main index in our evaluation.

Consequently, the AP and lateral talo-1^st^MT, AP talo-2^nd^MT, and HV angles were the four indices chosen. We used AP and lateral talo-1^st^MT angles and AP talo-2^nd^MT angle as surrogate indices for the progression of PV and the HV angle as surrogate for the progression of HV.

**Fig. 2:**
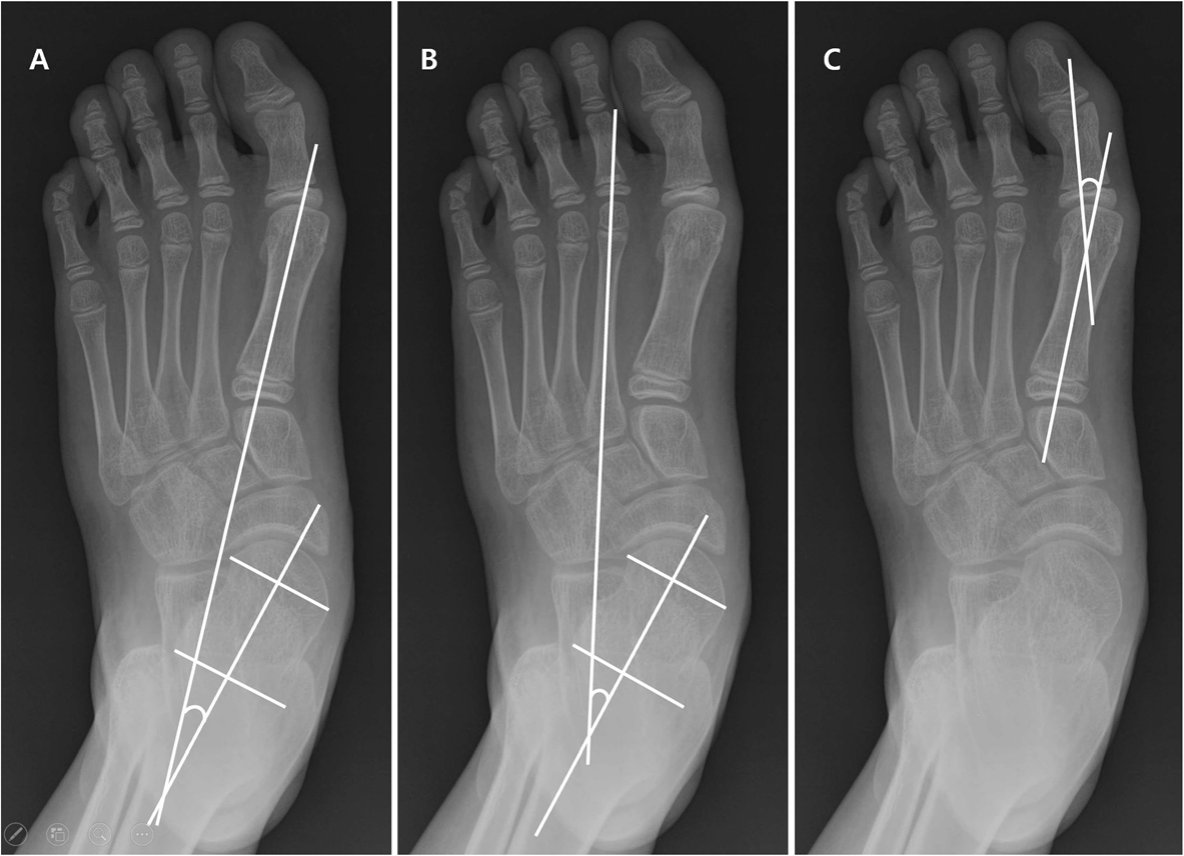
**a** The anteroposterior talo-first metatarsal angle is the angle between the line bisecting the longitudinal axis of the first metatarsal bone and the line bisecting the longitudinal axis of the talus on standing anteroposterior plain foot radiograph. **b** The anteroposterior talo-second metatarsal angle is the angle between the line bisecting the longitudinal axis of the second metatarsal bone and the line bisecting the longitudinal axis of the talus on standing anteroposterior plain foot radiograph. **c** The hallux valgus angle is the angle between the longitudinal axis of the first proximal phalanx and the longitudinal axis of the first metatarsus

**Fig. 3:**
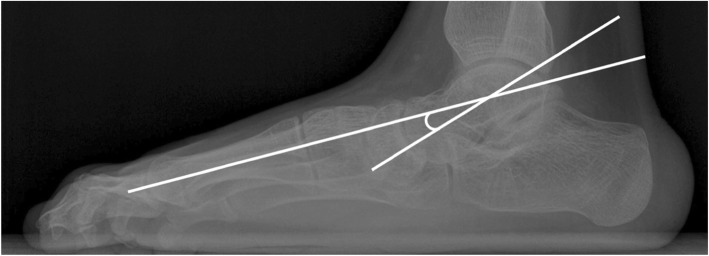
The lateral talo-first metatarsal angle is the angle between the line bisecting the longitudinal axis of the first metatarsal bone and the line bisecting the longitudinal axis of the talus on the standing lateral plain foot radiograph

The AP talo-1^st^ and talo-2^nd^MT angles are the angles between a line bisecting longitudinal axis of first and second metatarsal bones, respectively, and the line bisecting the longitudinal axis of the talus on standing AP plain foot radiograph [12] (Fig. 2). The lateral talo-1^st^MT angle is the angle between the line bisecting the longitudinal axis of the first metatarsal bone and the line bisecting the longitudinal axis of the talus in standing lateral foot radiograph [12] (Fig. 3). The HV angle is the angle between longitudinal axis of the first proximal phalanx and first metatarsus [12, 16] (Fig. 2).

After consensus building, reliability test was conducted before primary measurements. Sample size estimation showed that a minimum of 36 feet (18 left and 18 right) radiographs should be assessed. Three authors(JJM, KHS, and MSP) with 2, 16, and 19 years of orthopedic experience determined the interobserver reliability using intraclass correlation coefficients(ICCs), and independently measured the radiographs in a blinded fashion. Four weeks after the primary measurements, intra-observer reliability was assessed by one of the authors(JJM) who repeated the radiographic measurements.

Following reliability testing, two authors(JJM and MSP) measured indices in all the radiographs. Patient’s age, sex, GMFCS level, involvement(unilateral/bilateral), history of tendo-Achilles lengthening(TAL) or gastrocnemius recession(Strayer), laterality of the feet, and the date the radiographs were also included.

### Building a linear mixed model

The progression rate of the AP and lateral talo-1^st^MT, AP talo-2^nd^MT, and HV were adjusted by multiple factors using a linear mixed model, with sex, GMFCS, involvement (bilateral/unilateral), and TAL/Strayer as the fixed effects and laterality and each subject as random effects. The covariance structure was assumed as the variance components. The restricted maximum likelihood estimation was used to estimate parameters for the linear mixed model [17–19]. By examining the individual pattern of the annual changes in the AP and lateral talo-1^st^MT, AP talo-2^nd^MT, and HV angles as well as the follow-up duration, a linear mixed model with a random slope and a random intercept was suggested. The slope was the progression rate of the AP and lateral talo-1^st^MT, AP talo-2^nd^MT, and HV angles per year. Linear follow-up duration(age) effect was interpreted to evaluate the estimation of the measurements. The models were accepted as valid for estimating the responses using the Akaike information criterion(AIC) and the Bayesian information criterion(BIC). A smaller AIC or BIC value is preferred in terms of model selection [17–19].

### Statistical analysis

In this study, reliability was assessed with the use of ICCs and a two-way mixed-effect model, assuming a single measurement and absolute agreement [20, 21]. With the use of an ICC target value of 0.8, a Bonett approximation was employed in setting 0.2 as the width of 95% confidence intervals(CIs). The minimal sample size was calculated to be 36 [22].

Descriptive statistics were used to summarize patient demographics and radiographic measurements. The Kolmogorov-Smirnov test was used to verify the normality of the distribution of continuous variables. Descriptive statistics included the mean, standard deviation, and frequency. A linear mixed model was used to model the progression rates, assess the covariate effect, and examine the factors that contributed significantly to the rate of progression.

All statistical analyses were performed using the SAS Statistical package version 9.4(SAS Institute, Cary, NC, USA) and R version 3.5.1(R Foundation for Statistical Computing, Vienna, Austria; ISBN 3–900,051–07-0, URL http://www.r-project.org) with the stats package 2.3. All statistics tests were two-tailed, and CIs were considered significant when they did not include zero, and *p*-values < 0.05 were considered significant.

## Results

Seven hundred sixty-eight patients met the inclusion criteria. After implementation of inclusion and exclusion criteria, 194 patients were enrolled in this study (Fig. 1). The mean age and standard deviation of the patients at the time of their first visit to the outpatient clinic was 8.6 ± 3.1 years, with mean follow-up of 5.2 ± 4.0 years. Of the 194 patients, 90 were of GMFCS I, 70 GMFCS II, and 34 were GMFCS III. 111(57%) of 194 patients had history of TAL or Strayer at some point of their follow-up (Table 1).

**Table 1:** Summary of patient data (*n* = 194)

Parameters	Values
Patient information	
Sex (male/female)^a^	134/60
GMFCS level (I/II/III)^a^	90/70/34
Involvement (unilateral/bilateral)^a^	54/140
TAL/Strayer operation during follow-up^b^	111 (57%)
Age at initial assessment^c^	8.6±3.1 (2.2–17.3)
Duration of follow-up^c^	5.2±4.0 (1.0–15.0)
No. of follow-up visits^c^	2.3±0.7 (2–6)

*GMFCS* Gross Motor Function Classification System, *TAL* tendo-Achilles lengthening

^a^Data are given as the number of patients

^b^Data are given as the number (percentage) of patients

^c^Data are given as the mean, standard deviation, and range

The interobserver reliability ranged from 0.917 to 0.953, with AP talo-1^st^MT angle(ICC, 0.917), lateral talo-1^st^MT angle(ICC, 0.934), AP talo-2^nd^MT angle(ICC, 0.937), and HV angle(ICC, 0.953) (Table 2). The intraobserver reliability ranged from 0.931 to 0.961.

**Table 2:** Interobserver and intraobserver reliability of the radiographic measurements

	Interobserver reliability		Intraobserver reliability	
Measurement	ICC	95% CI	ICC	95% CI
AP talo-first metatarsal angle	0.917	0.810–0.961	0.944	0.893–0.971
AP talo-second metatarsal angle	0.937	0.888–0.966	0.931	0.869–0.964
Hallux valgus angle	0.953	0.920–0.974	0.961	0.925–0.980
Lat talo-first metatarsal angle	0.934	0.889–0.963	0.949	0.903–0.974

*ICC* intraclass correlation coefficients, *CI* confidence interval, *AP* anteroposterior, *Lat* lateral

**Fig. 4:**
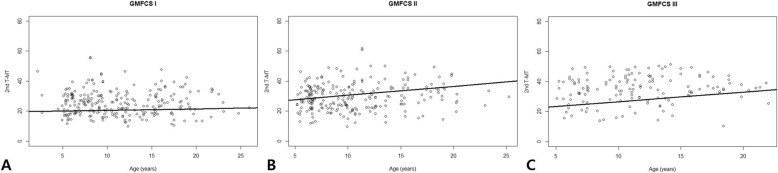
Scatterplots showing the progression of the talo-second metatarsal angles according to age for Gross Motor Function Classification System (GMFCS) levels I (**a**), II (**b**), and III (**c**). There is a positive correlation between the talo-second metatarsal angle and the follow-up duration for GMFCS levels II and III. The points represent all the foot radiographs obtained. The solid lines represent an estimation of the progression by a linear follow-up duration effect

In our mixed model, age was a major risk factor in GMFCS II and III patients with annual progression rate of AP talo-2^nd^MT angle of 0.59°(*p* < 0.0001) and 0.64°(*p* = 0.0007) respectively, whereas age was not significant(*p* = 0.3269) in GMFCS I patients (Table 3 and Fig. 4).

**Table 3:** Factors affecting the talo-second metatarsal angle according to GMFCS level

	Estimate	95% CI	*p* Value
GMFCS level I			
Intercept	19.64	15.38 to 23.90	<0.0001
Age	0.09	-0.10 to 0.29	0.3269
Sex (female)	-0.50	-3.85 to 2.84	0.7644
Involvement (bilateral)	2.63	-0.77 to 6.02	0.1273
No TAL/Strayer	4.00	1.18 to 6.83	0.0060
Side (left)	1.64	-0.15 to 3.44	0.0724
GMFCS level II			
Intercept	24.66	17.60 to 31.72	<0.0001
Age	0.59	0.31 to 0.88	**<0.0001**
Sex (female)	-0.37	-4.19 to 3.44	0.8470
Involvement (bilateral)	-0.95	-7.28 to 5.38	0.7664
No TAL/Strayer	-3.48	-6.73 to -0.24	0.0357
Side (left)	0.38	-0.94 to 2.30	0.4050
GMFCS level III			
Intercept	20.13	4.20 to 36.07	0.0151
Age	0.64	0.30 to 0.99	**0.0007**
Sex (female)	4.56	-0.89 to 10.01	0.0997
Involvement (bilateral)	5.26	-10.14 to 20.66	0.4985
No TAL/Strayer	-3.84	-12.12 to 4.43	0.3580
Side (left)	-0.04	-2.37 to 2.30	0.9751

The linear mixed model was used to estimate the factors affecting the talo-second metatarsal angle. The Akaike information criteria were 1699, 1582, and 948 for each model

*GMFCS* Gross Motor Function Classification System, *CI* confidence interval, *TAL* tendo-Achilles lengthening

The AP talo-1^st^MT angle increased annually by 0.48° (*p* = 0.0010) and 0.52° (*p* = 0.0036) respectively in GMFCS II and III patients, whereas it was not significantly affected by age(*p* = 0.9819) in GMFCS I patients (Table 4).

**Table 4:** Factors affecting the talo-first metatarsal angle according to the GMFCS level

	Estimate	95% CI	*p* Value
GMFCS level I			
Intercept	12.16	7.88 to 16.44	<0.0001
Age	-0.00	-0.20 to 0.19	0.9819
Sex (male)	-1.86	-5.09 to 1.38	0.2563
Involvement (bilateral)	2.80	-0.53 to 6.12	0.0977
No TAL/Strayer	4.23	1.36 to 7.09	0.0044
Side (left)	1.85	-0.00 to 3.70	0.0503
GMFCS level II			
Intercept	16.68	9.90 to 23.46	<0.0001
Age	0.48	0.20 to 0.76	**0.0010**
Sex (male)	-1.62	-5.24 to 2.01	0.3785
Involvement (bilateral)	-0.54	-6.64 to 5.57	0.8618
No TAL/Strayer	-3.10	-6.19 to -0.00	0.0500
Side (left)	1.18	-0.35 to 2.72	0.1296
GMFCS level III			
Intercept	12.63	-4.53 to 29.80	0.1430
Age	0.52	0.18 to 0.86	**0.0036**
Sex (male)	4.89	-1.02 to 10.79	0.1036
Involvement (bilateral)	4.94	-11.77 to 21.65	0.5578
No TAL/Strayer	-5.76	-14.69 to 3.17	0.2026
Side (left)	0.31	-1.91 to 2.54	0.7793

Linear mixed model was used to estimate factors affecting the talo-first metatarsal angle. The Akaike information criteria were 1708, 1562, and 941 for each model

*GMFCS* Gross Motor Function Classification System, *CI* confidence interval, *TAL* tendo-Achilles lengthening

HV was significantly affected by age in all three GMFCS groups, increasing by 0.48° (*p* < 0.0001), 0.66° (p < 0.0001), and 1.19° (p < 0.0001) in I, II, and III patients, respectively (Table 5).

**Table 5:** Factors affecting the hallux valgus angle according to GMFCS level

	Estimate	95% CI	*p* Value
GMFCS level I			
Intercept	6.34	3.38 to 9.31	<0.0001
Age	0.48	0.34 to 0.61	**<0.0001**
Sex (female)	2.29	-0.02 to 4.59	0.0515
Involvement (bilateral)	1.92	-0.44 to 4.27	0.1088
No TAL/Strayer	-0.71	-2.69 to 1.26	0.4758
Side (left)	-0.45	-1.70 to 0.82	0.4840
GMFCS level II			
Intercept	6.38	0.52 to 12.24	0.0332
Age	0.66	0.43 to 0.89	**<0.0001**
Sex (female)	4.45	1.12 to 7.78	0.0093
Involvement (bilateral)	-1.65	-7.08 to 3.79	0.5490
No TAL/Strayer	-0.94	-3.36 to 1.48	0.4406
Side (left)	0.32	-0.79 to 1.43	0.5693
GMFCS level III			
Intercept	2.03	-10.04 to 14.10	0.7334
Age	1.19	0.80 to 1.59	**<0.0001**
Sex (female)	1.55	-2.38 to 5.47	0.4346
Involvement (bilateral)	0.48	-11.22 to 12.18	0.9353
No TAL/Strayer	3.43	-3.92 to 10.78	0.3558
Side (left)	-0.21	-2.23 to 1.80	0.8325

Linear mixed model was used to estimate factors affecting the hallux valgus angle. The Akaike information criteria were 1525, 1449, and 908 for each model

*GMFCS* Gross Motor Function Classification System, *CI* confidence interval, *TAL* tendo-Achilles lengthening

The lateral talo-1^st^MT angle showed improvement with patients age. GMFCS I patients showed 0.43°(p < 0.0001) of improvement, and in level II patients showed improvement of 0.61°(p < 0.0001). Interestingly, there was no significant improvement in the lateral talo-1^stMT^ angle(*p* = 0.0535)in GMFCS III patients (Table 6).

**Table 6:** Factors affecting the lateral talo-first metatarsal angle according to the GMFCS level

	Estimate	95% CI	*p* Value
GMFCS level I			
Intercept	18.65	14.23 to 23.07	<0.0001
Age	-0.43	-0.62 to -0.25	**<0.0001**
Sex (female)	0.23	-3.60 to 4.06	0.9057
Involvement (bilateral)	2.42	-1.28 to 6.12	0.1951
No TAL/Strayer	-1.10	-3.92 to 1.72	0.4371
Side (**l**eft)	1.33	-0.50 to 3.16	0.1519
GMFCS level II			
Intercept	24.01	14.83 to 33.20	<0.0001
Age	-0.61	-0.88 to -0.34	**<0.0001**
Sex (female)	-0.36	-5.60 to 4.89	0.8923
Involvement (bilateral)	2.83	-5.47 to 11.14	0.4997
No TAL/Strayer	3.15	-0.81 to 7.12	0.1180
Side (left)	0.61	-1.34 to 2.56	0.5344
GMFCS level III			
Intercept	49.81	29.71 to 69.90	<0.0001
Age	-0.50	-1.01 to 0.01	0.0535
Sex (female)	1.86	-4.74 to 8.46	0.5766
Involvement (bilateral)	-14.52	-34.07 to 5.03	0.1433
No TAL/Strayer	8.20	-3.05 to 19.44	0.1506
Side (left)	-1.56	-4.43 to 1.30	0.2806

Linear mixed model was used to estimate factors affecting the lateral talo-first metatarsal angle. The Akaike information criteria were 1563, 1576, and 1049 for each model

*GMFCS* Gross Motor Function Classification System, *CI* confidence interval, *TAL* tendo-Achilles lengthening

The talo-2^nd^ MT angle was 4° higher in patients with GMFCS level I (*p* = 0.0044) and 3.10° lower in patients with GMFCS level II (*p* = 0.05) among those who did not undergo TAL or Strayer’s operation (Table 4). The talo-1^st^ MT angle was 4.23° higher in patients with GMFCS level I (*p* = 0.0044) and 3.10° lower in patients with GMFCS level II (*p* = 0.05) among those who did not undergo TAL or Strayer’s operation (Table 5).

## Discussion

In our longitudinal assessment of progression of PV in CP patients, we have demonstrated that the major risk factor in PV progression is the GMFCS level. The talo-1^st^MT and talo-2^nd^MT angles, which are surrogate indices of forefoot abduction, were significantly aggravated with age in GMFCS II and III patients (*p* < 0.0001, *p* = 0.0007). HV aggravated in all three groups (p < 0.0001), and the lateral talo-1^st^MT angle, a surrogate of planus, showed significant improvement in GMFCS I and II patients (p < 0.0001, p < 0.0001), whereas there was no significant improvement in GMFCS III patients.

It has been previously noted in a longitudinal study that idiopathic PV deformity improves annually until patients reach skeletal maturity; the AP and lateral talo-1^st^MT angle improved on average by 2.1° and 0.7° respectively annually until skeletal maturity [7] and rate of improvement decreases starting from age 10 [7]. Therefore, it had been recommended that any surgical procedure to correct the deformity, such as calcaneal lengthening [23], calcaneostop [24], and calcaneal-cuboid-cuneiform osteotomy [25], be postponed until child reaches 10 years old. In contrast, patients with CP forefoot abduction, as represented by the talo-2^nd^MT angle, not only showed no improvement(GMFCS level I), but rather progressed by 0.59°(*p* < 0.0001) and 0.64°(*p* = 0.0007) in patients with GMFCS II and III. It is therefore reasonable to believe that in patients with CP, correction of PV should be addressed at the time of single-event multilevel surgery.

Because HV also progresses with age in patients with CP, utilizing conventional talo-1^st^MT angle to assess PV may lead to underestimation of PV progression. Hence, we used the talo-2^nd^MT angle, which is more independent of the progression of HV.

The lateral talo-1^st^MT angle showed improvement in patients with GMFCS I and II. This implies that planus deformity may improve over time while forefoot abduction progresses. This may be the effect of the TAL or Strayer, which improve sagittal deformity. Furthermore, malrotated lever arm, which affects the inner ray of the foot, may have more adverse effects on the forefoot abduction than on the planus.

While discussing the clinical implications of the present study, it is crucial to address limitations. First, the study was retrospective by design. A uniform protocol was not implemented in all subjects; thus, patients differed in age at initial assessment, duration of follow-up, and number of radiographs taken during follow-up. However, a linear mixed model consisting of fixed and random effects was selected to adjust the repeated measures or longitudinal data that are an inherent limitation of this study design. Second, confounding variables such as achilles tightness and patients’ activity level could not be identified with our data. With our data, it is difficult to conclude that TAL or Strayer’s operation is a risk factor for changes in the talo-metatarsal angle. These limitations may be explored in future study in order to verify effects of such variables.

## Conclusion

In conclusion, the GMFCS level was the most important factor influencing the progression of radiographic indices in PV. The AP talo-1^st^ and talo-2^nd^MT angles progressed in patients with GMFCS II and III. Physicians should take this result into consideration when planning the timing of the surgery.

## Data Availability

The data set supporting the conclusion of this article is available on request to the corresponding author.

## Abbreviations

AP: Anteroposterior
CI: Confidence interval
CP: Cerebral palsy
GMFCS: Gross Motor and Functional Classification System
HV: Hallux valgus
PV: Pes planovalgus
TAL: Tendoachcilles lengthening
Talo-1st MT, talo-2nd MT angle: Talus-first metatarsal angle, talus-second metatarsal angle
